# Adltformer Team-Training with Detr: Enhancing Cattle Detection in Non-Ideal Lighting Conditions Through Adaptive Image Enhancement

**DOI:** 10.3390/ani14243635

**Published:** 2024-12-17

**Authors:** Zhiqiang Zheng, Mengbo Wang, Xiaoyu Zhao, Zhi Weng

**Affiliations:** 1 College of Electronic Information Engineering, Inner Mongolia University, Hohhot 010021, China; zqzheng@imu.edu.cn (Z.Z.); 32256155@mail.imu.edu.cn (M.W.); 32256113@mail.imu.edu.cn (X.Z.); 2 State Key Laboratory of Reproductive Regulation & Breeding of Grassland Livestock, Hohhot 010030, China; 3 Research Base for Dairy Farming Engineering and Full Mechanization of Equipment, Ministry of Agriculture and Rural Affairs, Hohhot 010018, China

**Keywords:** low light, cattle detection, image synthesis, image enhancement, team-training

## Abstract

This study proposes an image enhancement detection technique based on Adltformer (Adaptive dynamic learning transformer) team-training with Detr (Detection transformer) to improve model accuracy in suboptimal conditions, addressing the challenge of detecting cattle in real pastures under complex lighting conditions—including backlighting, non-uniform lighting, and low light. This often results in the loss of image details and structural information, color distortion, and noise artifacts, thereby compromising the visual quality of captured images and reducing model accuracy. To train the Adltformer enhancement model, the day-to-night image synthesis (DTN-Synthesis) algorithm generates low-light image pairs that are precisely aligned with normal light images and include controlled noise levels. The Adltformer and Detr team-training (AT-Detr) method is employed to preprocess the low-light cattle dataset for image enhancement, ensuring that the enhanced images are more compatible with the requirements of machine vision systems. The experimental results demonstrate that the AT-Detr algorithm achieves superior detection accuracy, with comparable runtime and model complexity, reaching 97.5% accuracy under challenging illumination conditions, outperforming both Detr alone and sequential image enhancement followed by Detr. This approach provides both theoretical justification and practical applicability for detecting cattle under challenging conditions in real-world farming environments.

## Simple Summary

In smart agriculture, most researchers have focused on cattle detection under ideal lighting conditions, producing favorable results. However, research on the non-ideal lighting conditions commonly observed in real-world rangeland environments is still limited. In this study, we propose a cattle detection method based on adaptive image enhancement that integrates an enhancement model and a detection model through team-training. This approach tackles challenges stemming from the differences between machine vision and human vision tasks, ultimately improving target detection performance in extremely low-light environments.

## 1. Introduction

In recent years, continuous advancements in computer vision and deep learning technologies have established livestock monitoring systems as a cornerstone of the livestock industry. These systems allow farmers to monitor livestock in real time, optimize production processes, and precisely track and manage individual animals, thereby improving production efficiency and economic returns. Accurate cattle detection is pivotal to these systems, forming the foundation for applications such as health monitoring, behavior analysis, and resource allocation. However, deploying robust and accurate cattle detection systems in real-world conditions poses significant challenges due to variability in environmental and lighting conditions [1,2].

Numerous studies have concentrated on enhancing the accuracy of cattle detection under controlled conditions. For example, Hu H et al. (2020) employed the Yolo detection algorithm for side-view cattle identification, achieving a high accuracy of 98.36% [3]. Xu B et al. (2021) improved detection across multiple viewpoints using a RetinaNet-based algorithm integrated with transfer learning [4]. Xing Y et al. (2022) introduced an enhanced SSD algorithm (SFM-SSD), leveraging shallow feature modules to boost real-time detection and feature extraction [5]. More recent efforts, such as those by Lu Y et al. (2023) and Weng Z et al. (2023), have employed advanced models like Yolov5 and Yolov7 to refine cattle detection, with mean average precision (mAP) values exceeding 98% in controlled settings [6,7]. Qiao Y et al. (2023) presented a method for cattle face detection using the Yolov5-ASFF model, achieving the accurate detection of various body parts (e.g., individual, head, and leg) with an average accuracy of 94.7% on a self-constructed dataset [8]. While these advancements have yielded remarkable results, they primarily focus on ideal lighting conditions, which are seldom representative of real-world pasture environments.

In practical ranching scenarios, various challenges, such as uneven lighting, shadows, backlighting, and low illumination, can complicate detection, resulting in significant information loss in images. These challenges highlight the need for robust methods capable of adapting to non-ideal lighting conditions. Recent studies have explored various approaches to tackling low-light detection. For instance, Chen C et al. (2018) developed an end-to-end model that bypasses traditional preprocessing steps, showing promising results in low-light conditions [9]. Sasagawa et al. (2020) integrated generative models with Yolo to enhance detection in short-exposure images [10], while Cui Z et al. (2021) introduced a multi-task automatic encoding transformer for robust detection in dark environments [11]. More recent innovations include Qi Y et al.’s (2023) adaptive attention mechanism and Yin X et al.’s (2023) PE-Yolo framework, both focusing on low-light detail enhancement [12,13]. Cai Y et al. (2023) utilized a transformer algorithm integrated with retina theory for dark–light enhancement in images, incorporating image enhancement as a preprocessing step for the detection task [14].

These studies show that detection performance can be enhanced through image improvements and necessary algorithmic modifications. However, most of the aforementioned algorithms focus solely on enhancing image quality or modifying feature extraction capabilities, thereby overlooking the specific objectives required for effective machine vision analysis. Due to the inherent differences between human and machine vision target objectives [15], such approaches result in only limited improvements in detection accuracy. Moreover, deploying these algorithms on edge devices is challenging due to their high parameter count and the difficulty of ensuring real-time performance. In response to these shortcomings, we propose a novel cattle detection method based on adaptive image enhancement, integrating both an image enhancement model and a detection model through team-training. Specifically, by adding the Adltformer image enhancement model pre-training weights as a pre-encoder in front of the Detr detection model backbone network, Adltformer can work with Detr on detection tasks. This integrated approach addresses the challenges posed by the differences between machine and human vision tasks, enhancing detection accuracy in extremely low-light conditions.

Our main contributions are summarized as follows:

1. Training a low-light image enhancement model is presented, constructing image pairs that are compatible with the training features is essential. This study introduces a day-to-night image synthesis algorithm to transform normal-light cattle images collected from real pastures into synthesized low-light images that are aligned with the original images with controlled levels of noise.

2. An adaptive dynamic learning transformer-based two-branch network frame-work for low-light image enhancement is presented. The framework maps sRGB images to the raw-RGB space using a local branch inverse mapping function, followed by the use of learnable transformer attention query vectors in the global branch to optimally adjust key ISP parameters. This ensures that the enhanced output images closely resemble those captured under normal lighting conditions.

3. This study proposes an image enhancement detection algorithm called Adltformer team-training with Detr (AT-Detr). This approach optimizes the entire network’s parameters through the team-training Adltformer and Detr to minimize the loss function for the detection task. Specifically, the algorithm enhances low-light images prior to training with the Detr model, improving the accuracy of cattle detection.

## 2. Materials and Methods

### 2.1. Image Dataset Construction

#### 2.1.1. Data Set Sources

**Figure 1:**
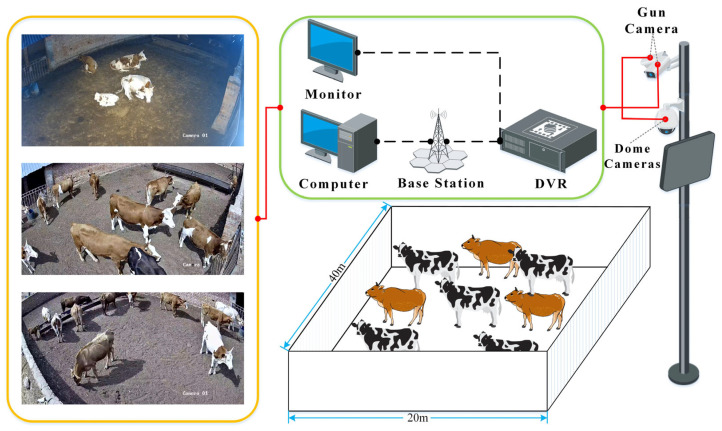
Schematic diagram of video data collection for cooperatives in Horinger County.

To capture images of cattle under varying lighting conditions, two 4-megapixel Hik-vision DS-2CD3T47EWD-L bullet cameras (Hikvision, Hangzhou, China) and one 2-megapixel Hikvision DS-2DC4223IW-D/GLT dome camera (Hikvision, Hangzhou, China) were installed at a designated breeding cooperative in Hohhot City and Horinger County, with all three cameras mounted on the same pole. The cameras store the collected video data in a Hikvision DS-7804N-F1/4P DVR (Hikvision, Hangzhou, China) and transmit the data back to the laboratory via a 4G signal provided by the base station [16]. The client computers access the required data through the network. The video data collection process is illustrated in Figure 1.

The video data were collected over three days and downloaded every half hour via a computer client, with each segment approximately 5 min in length. Excluding periods of corrupted data that could not be downloaded, 272 videos were obtained, consisting of 177 daytime videos and 95 nighttime videos. The compositions of the collected video data are shown in Table 1.

#### 2.1.2. Data Preprocessing

**Figure 2:**
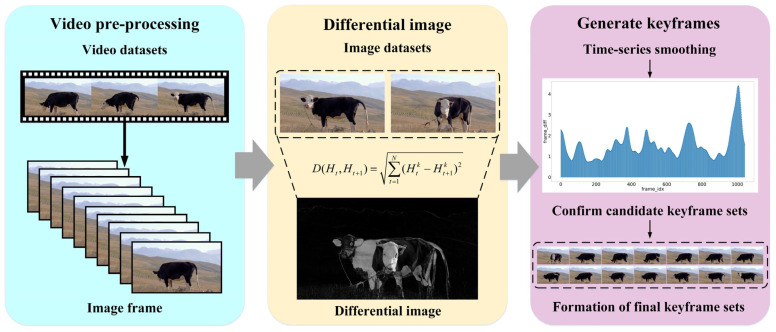
Flowchart of key frame extraction.

During the data collection process, a large amount of redundant information is often captured, and the relatively inactive daily activities of cattle result in significant data duplication. Therefore, data preprocessing is necessary before constructing a dataset. Extracting keyframes from ranch video captures a set of images that exhibit the greatest differences in cattle postures across frames, eliminating temporal redundancy and maximizing crucial information within the video. In this study, an FFmpeg-based library is employed for video key frame extraction; consecutive frames are differenced to compute the average pixel intensity, determining the magnitude of image changes. Typically, three key frame extraction methods are based on interframe differencing: differential intensity order, differential intensity thresholding, and local maxima. Local maxima extraction enhances image diversity and visual quality; while applying smoothing to the average interframe differential intensity time series can effectively reduce noise interference and prevent the erroneous identification of multiple frames as keyframes in similar scenes, thereby improving the accuracy and reliability of image processing. The key frame extraction process is illustrated in Figure 2.

**Figure 3:**
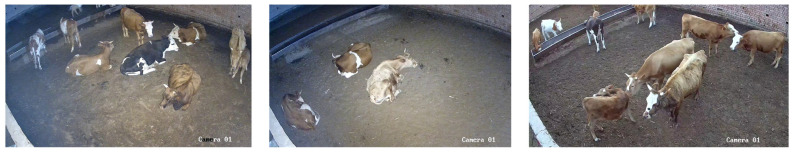
Images from different periods.

Key frame extraction eliminates a substantial number of duplicate images. However, the absence of cattle or the presence of excessively blurred images can significantly impact the subsequent detection tasks, necessitating a combination of manual and algorithmic cleaning to remove poor-quality images and those without cattle. After key frame extraction and data cleaning, 1688 normal-light images and 865 low-light images were retained, resulting in 2553 high-quality images. Figure 3 illustrates the images captured during different periods.

Given that no low-light data perfectly aligned with the normal-light cattle images, the 1688 normal-light cattle images were randomly divided into two subsets. The first subset contained 865 normal-light images, matching the number of real low-light images collected. The remaining 823 normal-light images were used to synthesize low-light images with the DTN-Synthesis algorithm. The specific categorizations and roles of the datasets are detailed in Table 2.

### 2.2. DTN-Synthesis Method

**Figure 4:**
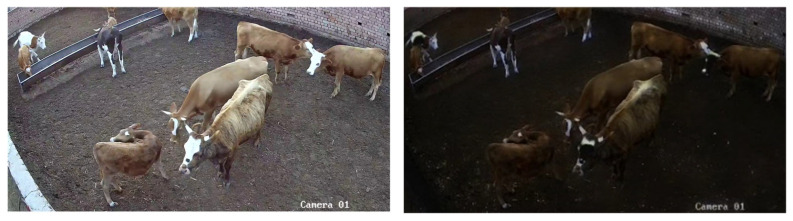
Nighttime images synthesized by the DTN-Synthesis algorithm.

Synthesizing nighttime image pairs that align with daytime images is a critical task in computer vision and image processing. In real-world scenarios, the differences in lighting conditions between day and night can result in significant variations in brightness, color, contrast, and other visual attributes. Nighttime images in particular often suffer from a lack of sufficient detail due to low light levels or noise. Many machine vision tasks require systems to process images captured under both daytime and nighttime conditions. However, compared with daytime image data, nighttime image data are generally scarce. Additionally, image enhancement training tasks require a large-scale dataset of paired images: noisy nighttime images are captured using short exposures and high ISO settings, and low-noise daytime images are captured with long exposures and low ISO settings processed through a neural image signal processor [17,18]. Capturing such image pairs requires perfect alignment, which can lead to variations in image geometry and poses due to the movement of cattle. Even minor offsets in camera positioning and angle can cause discrepancies between the images due to differences in the positioning and the perspectives of the cameras. To address this issue, this study introduces the DTN-Synthesis algorithm, which can generate both noisy and noise-free images. A synthesized nighttime image is illustrated in Figure 4.

**Figure 5:**
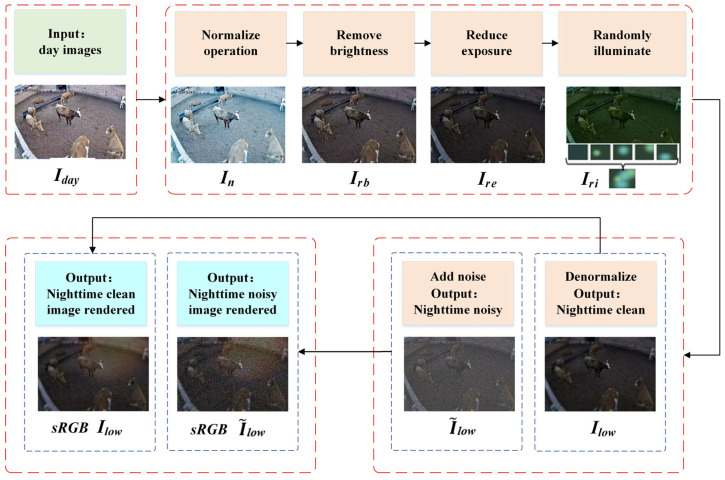
Algorithm synthesis process framework.

The synthesis process primarily involves reducing the brightness of the real daytime image, lowering its exposure level, simulating nighttime light sources to randomly illuminate the scene, and adding a controlled amount of noise to replicate a realistic nighttime image, ultimately generating either a noisy or noise-free nighttime image. The framework diagram of the algorithm’s synthesis process is depicted in Figure 5. It is assumed that the input daytime image is clear and free of noise.

We use $I_{day}\in R^{\frac{H}{2}\times \frac{W}{2}\times 4}$ to represent a clear and noise-free daytime image, where H and W denote the height and width of the image, respectively, in pixels. Firstly, the black and white levels of the input daylight image are adjusted, and the data is normalized. The normalized image is $I_{n}=(I_{day}-b_{l})/(w_{l}-b_{l})$, where $b_{l}$ and $w_{l}$ denote the black and white levels provided by the camera metadata.

By applying the white balance correctly when shooting images in normal outdoor light conditions, one can minimize the errors that occur during overall color correction.

For this purpose, we remove daylight illumination from the input image by applying a white balance. The white-balanced image is $I_{rb}=I_{n}\cdot L_{day}$, where $L_{day}=diag(\frac{1}{r},\frac{1}{g},\frac{1}{g},\frac{1}{b})$, and the green channel value, g, in $L_{day}$ is usually normalized to 1.

Compared with daytime images, nighttime images have low overall brightness and are illuminated by light sources of varying brightness. The exposure of the image is reduced using $I_{re}={k\cdot I}_{rb}$, where k is the average luminance scale factor of the night image. To imitate light sources at night to re-illuminate the scene in the image, 5 to 8 bright spots are randomly generated from a library of light sources collected from different night images to simulate night lights. The re-lit image is shown in Equation (1).

(1) $$I_{ri}=\frac{\sum_{i=1}^{N}\;I_{re}L_{{low}_{i}}⊙c_{i}D_{i}}{\sum_{i=1}^{N}\;c_{i}D_{i}}$$

Here, $L_{{low}_{i}}=diag(r_{i}，g_{i}，g_{i}，b_{i})$ denotes the set of samples in the library of nighttime light sources, ⊙ is an element-by-element multiplication, and $c_{i}$ is a scalar to control the illumination level of each light source. A 2D Gaussian function is constructed using $D_{i}=d\cdot G(x_{i},y_{i},\sigma_{x_{i}},\sigma_{y_{i}})$ to determine the number of light sources and their locations, where d represents the number of light sources, and $\left(x_{i},y_{i}\right)$ represents the position of the center of the light source; the position is restricted to the image at a distance of 15% from the boundary. The diffusion of the light source is regulated by ${(\sigma }_{x_{i}},\sigma_{y_{i}})$, and its diffusion range is half of the image size. Afterward, $I_{ri}$ is inverse-normalized to obtain the final synthesized night image, $I_{low}=I_{ri}\cdot \left(w_{l}-b_{l}\right)+$ $b_{l}$.

The image, $I_{low}$, represents a low-noise night image with long exposure and low ISO gain, which can be rendered directly at this stage to produce the final clean and low-noise night image, $sRGBI_{low}$. If a synthetic image comparable to a real night image is to be obtained, appropriate noise must be added to $sRGBI_{low}$ to produce a noisy night image with short exposure and high ISO gain. To align the noise added to the image with the actual application scenario and to describe the real characteristics of the data more accurately, the recognized heteroskedastic Gaussian model [18] is used. The generated noisy raw image is shown in Equation (2):

(2) $${\tilde{I}}_{low}=I_{low}+N(0,\partial_{1}I_{low}+\partial_{2})$$

where $\partial_{1}$ is a shooting noise parameter, introduced by the process of the image being captured or acquired, and $\partial_{2}$ represents a reading noise parameter, introduced by the image being read or transmitted to the processing unit. $\partial_{1}\;and{\;\partial }_{2}$ are set to different empirical values based on clean low-noise and true high-noise night image pairs. The noise-added ${\tilde{I}}_{low}$ image is rendered to obtain a synthetic night image, $sRGB{\tilde{I}}_{low}$, which is similar to a noisy night image taken with a short exposure and high ISO gain.

### 2.3. Adltformer Method

#### 2.3.1. Motivation

When a photo is taken under suboptimal lighting conditions, light is reflected from the scene into the camera lens and subsequently reaches the image sensor. The sensor converts the captured light into electrical signals, which are then processed by an image signal processor (ISP) [19,20] to generate a digital image that is stored on the camera’s storage medium.

The objective of image enhancement is to transform an image, $I_{low\;}$, captured under suboptimal lighting conditions, to resemble an image, $I_{day}$, acquired under ideal lighting conditions. Previous approaches typically achieved this transformation between $I_{low\;}$ and $I_{day}$ using end-to-end networks or by learning high-level representations (such as illumination maps [21], color transform functions [22], 3D-LUTs [23], among others). However, actual luminance degradation occurs at the raw-RGB stage, and the ISP processing within the camera involves complex nonlinear operations. Consequently, many researchers have focused on the raw-RGB stage rather than the sRGB stage [18].

To better learn and optimize key parameters (e.g., white balance, color correction matrix, and gamma correction) during ISP processing, the image brightness can be dynamically adjusted [24], thereby generating an image that closely resembles one captured under normal lighting conditions. Brooks et al. [19] reversed each key step in the ISP processing pipeline, ultimately converting the input sRGB image back into unprocessed raw-RGB data. Subsequently, Afif et al. [25] mapped the sRGB image onto its corresponding raw-RGB space using an inverse mapping function, as represented by the conversion equation in Equation (3). Here, f denotes the network encoder that maps $I_{low}$ to its corresponding raw-RGB image, while $g_{t}$ maps $f(I_{low})$ to a separate encoder for the target image, 

.$I_{day}$

(3) $$I_{day}=g_{t}(f\left(I_{low}\right))$$

The function $f\left(\cdot \right)$ processes the sRGB image using a pixel-level least squares method. $f\left(\cdot \right)$ comprises two main branches: one branch predicts the pixel-level multiplicative component, $I_{M}$, while the other predicts the additive component, $I_{A}$. The equation for the least squares processing is provided in Equation (4).

(4) $$f(I_{low})=I_{low}⊙I_{M}+I_{A}$$

The $g_{t}(\cdot )$ function is inspired by the target detection algorithm, Detr [26], in which different target objects are managed using the transformer query mechanism. Thirteen distinct queries are used to dynamically adjust parameters in $g_{t}$ that are related to ISP operations. The query parameters are optimized through continuous iterations during training to align $I_{low}$ more closely with the target image, $I_{day}$. A simplified representation of the ISP process in $g_{t}(\cdot )$ is provided in Equation (5).

(5) $$g_{t}(\cdot )=Gamma(M_{ccm}(M_{wb}(\cdot )))$$

White balance (WB) typically constitutes the first stage of the ISP processing pipeline. $M_{wb}$ is a 3 × 3 diagonal matrix used to transform the RGB channels linearly to better match the colors in natural lighting conditions. The color correction matrix (CCM) typically constitutes the second stage of the ISP processing pipeline, corresponding to the RGB domain stage. $M_{ccm}$ is a 3 × 3 matrix primarily used to convert image data post-white balance processing into the standard RGB color space. Gamma correction typically constitutes the third stage of the ISP processing pipeline. After CCM processing, RGB data are still linear and require a gamma-based nonlinear transformation to render the image closer to the visual characteristics of the sRGB color space, enhancing display accuracy. In summary, the Adltformer image enhancement formula is presented in Equation (6). γ denotes the gamma correction parameter, which is individually controlled using a query.

(6) $$I_{day}=(max(\sum {{\;M}_{wb}M}_{ccm}(I_{low}⊙I_{M}+I_{A})),0{)}^{\gamma }$$

#### 2.3.2. Model Structure

**Figure 6:**
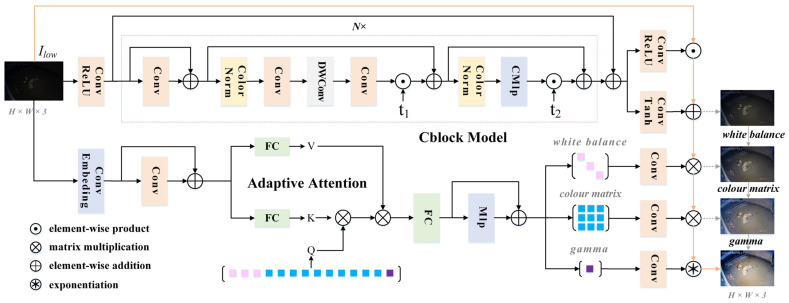
Image enhancement model network structure diagram.

It is widely accepted that both local and global information are crucial for effective visual scene understanding. Building on this insight, our approach integrates the strengths of both paradigms, allowing for the direct modeling of local and global dependencies. The Adltformer network is structured into two distinct branches: the local branch, f, which performs inverse mapping from sRGB to raw-RGB, and the global branch, g, which dynamically optimizes key ISP parameters. The complete network architecture of the algorithm is illustrated in Figure 6. Given an sRGB low-light image, $I_{low}$, as input, the target image, $I_{day}$, is predicted after processing through the local and global branches.

Local Branch: The local branch primarily comprises two independent sub-branches responsible for predicting the pixel-level multiplicative image, $I_{M}$, and the additive image, $I_{A}$, where each sub-branch comprises N-many Cblock modules. To better preserve image details and maintain consistency between input and output image resolutions, we propose a network architecture based on transformers. Unlike other popular architectures, our structure accommodates arbitrarily sized images as inputs while preserving the original image resolution in the output.

First, the low-light image, $I_{low}$, is processed through an initial 3 × 3 convolutional layer with an activation function to expand the channel dimensions, followed by two independent branches, each consisting of N-many Cblock models, where N is set to 3 in this study. Next, the residuals between the initial input image features and the output features from the Cblock models are combined through element-wise addition to further preserve the original image details. Each of the final two independent branches is passed through a 3 × 3 convolutional layer to reduce the channel dimensions, and the ReLU or Tanh function is applied to produce the final predicted pixel-level multiplicative component, $I_{M}$ and additive component, $I_{A}$.

The Cblock model is the primary module within the local branch, with its main function being to enhance local image details. The Cblock model initially encodes positional information using a 3 × 3 position-embedded convolutional layer, which is then summed with the original input. Next, a custom color normalization layer [27] performs both linear and color transformations on the input tensor, incorporating two learnable parameters that are dynamically adjusted during training to optimize model performance. Subsequently, three convolution operations—PWConv, DWConv, and PWConv—are performed, and the resulting feature map is element-wise multiplied by the learnable parameter, $t_{1}$ [24]. The original input features are added to the result of the multiplication and are then fed back into the color normalization layer. To simplify the design and better capture the nonlinear relationships between inputs and outputs, enabling the model to handle more complex tasks, we employ CMLp, a hybrid structure combining convolutional layers and MLp [24]. Finally, to improve model convergence, the processed results are element-wise multiplied by $t_{2}$ to complete the module [28].

Global branch: The global branch primarily employs adaptive attention to predict the WB, CCM, and gamma parameters, which regulate the global characteristics of the image. Using these key parameters, the local component, $f(I_{low})$, is processed through ISP correction, enabling the low-light image to more closely resemble one captured under normal lighting conditions, achieving image enhancement.

First, the low-light image, $I_{low}$, is fed into the conv_embedding module and processed using a 3 × 3 convolution for dimensionality reduction, enabling the model to encode high-dimensional features at a lower resolution. The output features are then passed to the adaptive attention module, where the image features undergo dimensional transformation and feature expansion through the fully connected layer (FC) to compute the attention mechanism. Afterward, the attention mechanism is applied to the normalized features, followed by an MLp convolution operation. This process generates thirteen parameters, representing a 3 × 3 white balance diagonal matrix, a 3 × 3 color matrix, and one-dimensional gamma values. These parameters correct the local branch output image features, completing the low-light image enhancement task.

Leveraging the transformer’s strength in capturing global information, the adaptive attention module primarily conducts adaptive dynamic learning with the images’ global features. Inspired by the Detr network for target detection, we utilize a randomized query to interact with the key and value generated from the image itself, without incorporating a multi-head attention mechanism. Using a dynamic query-learning strategy, the network adaptively adjusts the white balance diagonal matrix, color correction matrix, and gamma values to modify the image’s global characteristics as training epochs progress. Notably, the designed ISP parameters are optimally adjusted for each image, effectively assigning a unique value to perform the low-light image enhancement task on a per-image basis.

**Figure 7:**
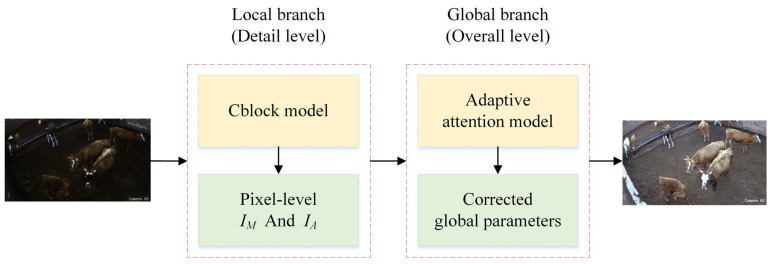
Interaction diagram between local and global branches.

Local and global branch interactions: The primary task of the local branch is to enhance image details at the pixel level by predicting the pixel-level multiplication component ($I_{M}$) and addition component ($I_{A}$). These components enhance the local details of the image while preserving the original resolution of the input image. The global branch, on the other hand, improves the overall appearance of the image by adjusting ISP parameters, such as the WB, CCM, and gamma, bringing the image closer to its normal-light counterpart. In general, the image details produced by the local branch are refined by the parameters generated by the global branch, ensuring that the overall visual quality of the image is optimized while preserving the local details. The interactive relationship between the local and global branches is illustrated in Figure 7.

### 2.4. AT-Detr Method

**Figure 8:**
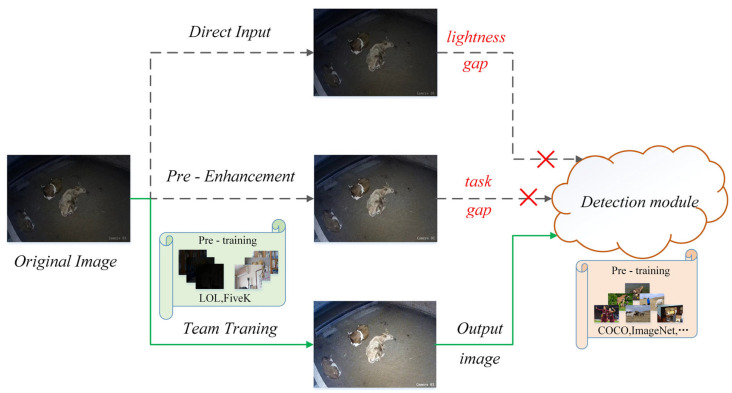
Three methods for low-light image detection.

For cattle detection under non-ideal lighting conditions, as illustrated in Figure 8, most current detection frameworks are trained on extensive normal-light datasets, yielding high-performing models that are subsequently applied to low-light image data. However, low-light images lead to poor model performance due to the loss of image details and structural information, color distortion, and noise artifacts. Furthermore, using image enhancement methods to preprocess cattle images for subsequent detection tasks creates discrepancies between the objectives, as image enhancement focuses on improving image quality (e.g., PSNR and SSIM), while detection targets machine vision accuracy (e.g., mAP and recall) [24].

**Figure 9:**
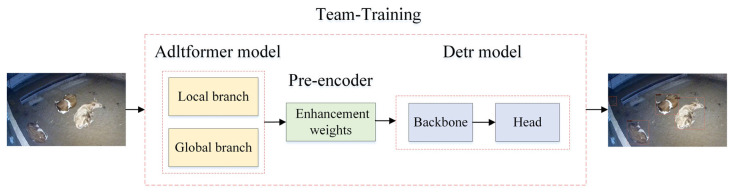
AT-Detr workflow diagram.

To address these issues, we propose a method for team-training the Adltformer enhancement model and the Detr detection model by incorporating the Adltformer enhancement model’s pre-trained weights as a pre-encoder preceding the Detr backbone network, enabling Adltformer to collaborate with Detr for detection tasks. The workflow of the AT-Detr algorithm is illustrated in Figure 9. Upon inputting a low-light image, the model first passes through the Adltformer image enhancement algorithm, which trains the weights and generates an enhanced image. Subsequently, the enhanced image is fed into the Detr detection model, where it undergoes further training to obtain the detection weights for the low-light image detection task.

During the training process, to ensure the enhanced image is more aligned with the machine vision target, the loss function of the Detr detection model is minimized by optimizing the parameters of the entire network, as represented in Equation (7):

(7) $$\underset{i\in I,d\in D}{min}\;L_{obj}(\hat{t},t)$$

where $\hat{t}$ denotes the predicted value of the Detr detection image, and t denotes the baseline true value. $L_{obj}$ denotes the target detection loss. i∈I,d∈D denote the image traversal enhancement task and the detection task, respectively.

For the input image, x, after the enhancement task, $I(I_{1}(x))$, the processed $I_{t}(x)$ is obtained, the $I_{t}(x)$ is passed through the detection task, $D(I_{t}(x))$, to finally obtain the predicted value, $\hat{t}$. The process is shown in Equation (8).

(8) $$I_{t}(x)=I(I_{1}(x)),\hat{t}=D(I_{t}(x))$$

## 3. Experimental Results and Analyses

To validate the effectiveness of the AT-Detr algorithm in detecting cattle under low-light conditions, this section presents image enhancement experiments using the Adltformer algorithm, image detection experiments using the AT-Detr algorithm, and relevant comparison experiments.

### 3.1. Experiment Setup

Evaluation Datasets: This section utilizes the cattle dataset collected by the research team as the primary experimental dataset, comprising 3376 images captured under various lighting conditions. This dataset includes 1688 normal-light images, 865 low-light images, and 823 synthesized low-light images. Additionally, the publicly available ExDARK dataset was employed as a supplementary experiment. This dataset’s primary feature is its extensive collection of 7363 low-light images of real-world scenes, spanning 10 different lighting conditions ranging from extremely low-light environments to dusk. Although it does not contain cattle-specific images, this dataset is well-suited for evaluating the algorithms’ performance in detecting objects under low-light conditions.

Implementation Details: Extensive experiments were conducted on a Linux system (kernel version 5.15.0-101-generic) running Ubuntu 20.04.1 on a host computer equipped with an i9-9900K processor, 32 GB of RAM, and an RTX 6000 graphics card. Various data augmentation techniques (e.g., random rotation, scaling by a factor of 0.65–1.35, and random flipping) were applied to improve the model’s performance. The Adam optimizer was employed to train the detection model, with an initial learning rate and weight decay set to 0.0001 to mitigate model overfitting. The STEP learning rate strategy was implemented, adjusting the learning rate at specific training intervals.

Evaluation Metrics: The most widely used metrics for assessing image quality are the peak signal-to-noise ratio (PSNR) and structural similarity index (SSIM) [29], which are primarily utilized to measure the similarity between the original image (or video frame) and the processed image. To evaluate the detection performance and image enhancement efficiency of the model, the precision (P), recall (R), mean average precision (mAP), average precision (AP), frames per second (FPS), and number of parameters (Params/M) are used as evaluation metrics.

### 3.2. Image Enhancement Experiment

#### 3.2.1. Adltformer Experiment

Before training the Adltformer model, the algorithm evaluated the PSNR and SSIM metrics between the synthesized low-light images and the normal-light images. The results are presented in Table 3.

Subsequently, the Adltformer model was trained using image pairs, and the pre-trained weights were obtained. The synthesized low-light dataset was then enhanced using these weights, and the PSNR and SSIM image quality metrics were reevaluated. The results are presented in Table 4.

Table 2 and Table 3 show that the PSNR and SSIM metrics significantly improved after applying image enhancement using the Adltformer model compared with the values obtained before enhancement. Specifically, the PSNR value increased to 17.254, 7.026 higher than before enhancement, while the SSIM value reached 0.699, showing an improvement of 0.344.

#### 3.2.2. Comparison of Image Enhancement Algorithms

Using the same dataset and testing environment, the Adltformer model was compared with other state-of-the-art (SOTA) image enhancement algorithms in a comparative experiment to evaluate its image quality metrics (PSNR and SSIM) and algorithm efficiency metrics (FPS and Params). The results for the image quality and algorithm efficiency of various enhancement algorithms are presented in Table 5.

**Figure 10:**
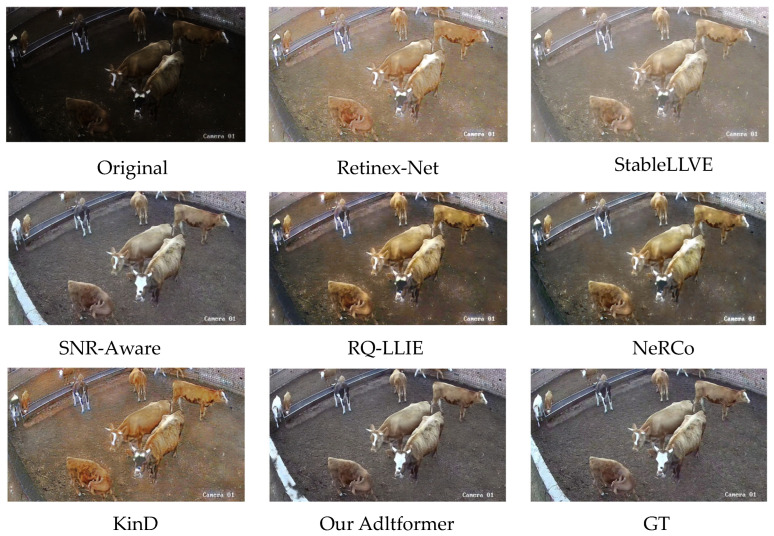
Enhancement effect of different algorithms.

By analyzing the relevant metrics, the Adltformer model can demonstrate superior performance in terms of both image quality and model efficiency compared with existing SOTA methods. The qualitative results after enhancement with different algorithms are illustrated in Figure 10. The Adltformer model is visually closest to the reference image, while the SNR-Aware model ranks second but exhibits noticeable image blurring.

### 3.3. Image Detection Experiment

To evaluate the impact of the three methods illustrated in Figure 8 on the accuracy of low-light image detection, the following three experiments were designed. First, an original image is directly fed into the Detr model without any preprocessing or enhancement to assess the baseline detection performance of the model. Second, real low-light images are preprocessed using Adltformer and other enhancement models, and the resulting images are fed into the Detr model to analyze the detection performance after image enhancement. Finally, the Adltformer and Detr detection models were jointly trained by integrating the Adltformer as a pre-encoder in front of the Detr algorithm’s backbone network using image enhancement pre-trained weights to determine whether the AT-Detr algorithm could further enhance the detection performance under low-light conditions.

#### 3.3.1. Detr-Based Experiment

The Detr algorithm was trained using 865 real low-light images, 865 normal-light images, and 1730 mixed low-light and normal-light images without applying any enhancement processes. The experimental results are presented in Table 6.

The table shows that the Detr model demonstrates the poorest detection performance on mixed images and the highest recall and mAP50 scores on normal-light images, while its overall performance on real low-light images is intermediate. A comparison of the three datasets verifies that the inclusion of low-light images results in reduced detection accuracy.

#### 3.3.2. Comparison of Image Detection Algorithms

To verify the detection performance of the AT-Detr team-training, a series of comparative experiments were conducted using multiple algorithm models on the same low-light real image dataset. To ensure the reliability and validity of the results, the experimental parameter settings, as well as the hardware and software environments, were kept consistent. The specific experimental results are presented in Table 7.

These results indicate that the AT-Detr algorithm proposed in this study demonstrates substantial improvements in recall and mAP50 metrics. Compared with using Detr alone for detection, most algorithms show superior performance after image enhancement; however, a few algorithms perform worse than Detr. This performance drop may be due to unrealistic enhancement, leading to a loss of image details, color distortion, noise retention or amplification, and the presence of artifacts in the enhanced image. This observation further confirms that low-light image enhancement for advanced vision tasks must be aligned with the task’s specific goals to avoid suboptimal results.

The proposed AT-Detr algorithm ensures that the enhanced image is more aligned with the machine vision objectives while maintaining high-quality enhancement. Compared with Detr, the proposed algorithm achieves a recall of 99.4%, reflecting a 1% improvement, and an mAP50 of 97.5%, indicating a 1.1% increase. An increased recall rate detects more targets, though it may also lead to some false detections; conversely, a higher mAP50 indicates more precise positioning and classification for the detected targets. Simultaneously improving both indicators allows the model to function more stably in low-light environments, reducing both missed and false detections while enhancing detection and positioning accuracy. This improvement is essential for practical applications such as pasture management and health monitoring.

**Figure 11:**
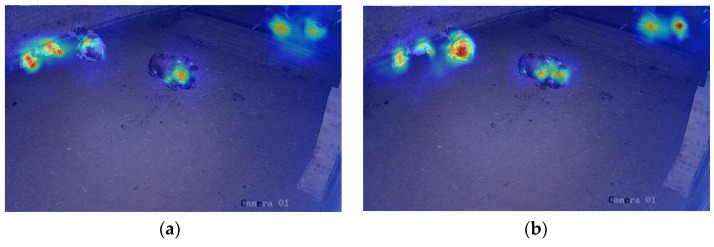
Comparison of the heat map effects of Detr and AT-Detr: (**a**) Detr model; (**b**)AT-Detr model.

To visualize the attention distribution of Detr and AT-Detr across different regions of the image, this study evaluates the target detection capabilities of the two models in low-light environments using heat maps. In Figure 11, a heat map of Detr is displayed on the left, while a heat map of AT-Detr is shown on the right. As shown, Detr can focus on the target in normal regions, but its attention is weaker than that of AT-Detr. Moreover, Detr struggles to distinguish individual targets effectively when they are closely clustered. In edge regions, low-light conditions exacerbate image blurring, noise, and color distortion, causing Detr to fail to accurately detect target individuals, leading to a higher incidence of missed and false detections. By contrast, AT-Detr significantly improves target detection in edge regions under low-light conditions by enhancing local details, improving overall image quality, and optimizing network parameters through team-training. Consequently, AT-Detr achieves superior detection accuracy.

**Figure 12:**
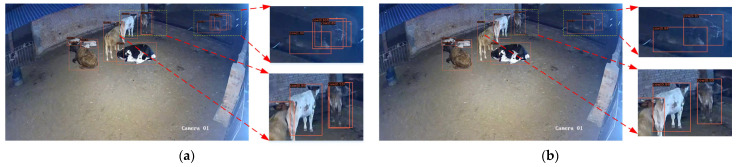

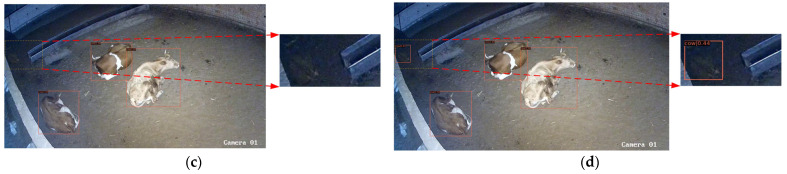
Comparison of Detr (left half) and AT-Detr (right half) detection effects: (**a**,**c**) Detr alone; (**b**,**d**) AT-Detr algorithm. In order to better observe the detection effect, the yellow boxes part in the figure is enlarged.

The qualitative results for the AT-Detr algorithm are illustrated in Figure 12. The left half presents the detection results using Detr alone, while the right half displays the results for the AT-Detr algorithm. Important areas are magnified to highlight specific details in the image. In comparison, the Detr algorithm struggles to accurately detect cows or misses some entirely due to underexposure in the image. By contrast, the AT-Detr algorithm correctly detects the target cows, demonstrating superior performance in low-light cow detection.

#### 3.3.3. Public Dataset Experiments

To verify the utility and generalization capability of AT-Detr, comparative experiments were conducted on the publicly available ExDARK dataset [45] using the Detr model along with various image enhancement algorithms. The results for various enhancement algorithms across different ExDARK categories are presented in Table 8.

The data indicate that the AT-Detr algorithm achieves the highest accuracy for four of the detection categories (Bicycle, Car, Cat, and People). To evaluate the overall detection performance of the algorithm, the accuracies for different categories were summed and averaged to compute an mAP50 score, as shown in the last column of Table 8. The AT-Detr algorithm achieves an mAP50 of 72.8% on the ExDARK dataset, 0.3% higher than the recent StableLLVE self-supervised learning algorithm and 1.9% higher than the RUAS zero-order learning algorithm.

**Figure 13:**
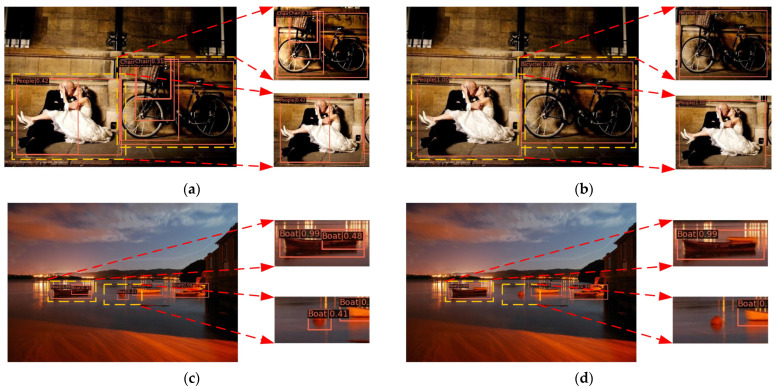
Comparison of Detr (left half) and AT-Detr (right half) detection effects: (**a**,**c**) Detr alone; (**b**,**d**) AT-Detr algorithm. In order to better observe the detection effect, the yellow boxes part in the figure is enlarged.

Figure 13 compares the detection results between the Detr algorithm and the AT-Detr algorithm on the publicly available ExDARK dataset. The left half shows the detection results for the Detr algorithm, while the right half presents the results for the AT-Detr algorithm. The Detr algorithm exhibits issues such as incorrect or missed object detection, whereas the AT-Detr algorithm detects target objects accurately and comprehensively. This further confirms that the proposed algorithm offers significant reference value for advanced vision tasks.

### 3.4. Ablation Studies

To evaluate the effectiveness of the Adltformer image enhancement algorithm and its team-training approach, ablation experiments were conducted under consistent dataset and parameter conditions to assess the impact of each module on the performance of both the image enhancement and object detection algorithms [48].

The evaluation results for the Adltformer image enhancement algorithm are presented in Table 9. In the local branch, after replacing the original Layer Norm with the Color Norm designed in this study, PSNR and SSIM were improved by 2.941 and 0.102, respectively. This improvement indicates that, by performing linear and color transformations on the input image, along with incorporating two learnable parameters that dynamically adjust during training, the model can better preserve the original image’s detailed information, optimizing its performance. In the global branch, we first evaluated the effectiveness of the adaptive attention model, which was enhanced based on the Detr detection network. Subsequently, we employed randomized queries to interact with the key and value generated by the image itself and introduced ISP parameters, such as WB, CCM, and gamma values, to regulate the global image information. This confirmed the enhancement of the image by adding various ISP parameters. Specifically, after incorporating the adaptive attention model, PSNR and SSIM improved by 5.702 and 0.281, respectively, leading to a significant qualitative improvement and confirming the effectiveness of this module.

The evaluation results of the team-training between the Adltformer image enhancement algorithm and the Detr target detection model are presented in Table 10. On the low-light cow dataset, compared with the original Detr, training with images enhanced by the Adltformer local branch resulted in improvements of 0.4% and 0.3% in recall rate and mAP50, respectively. This indicates that enhancing the local detail information of the image helps improve detection accuracy in low-light conditions. Building on this, detection accuracy was further improved after incorporating the global branch, which adaptively adjusts the overall ISP parameters of the image. Specifically, the recall rate and mAP50 increased by 0.7% and 0.6%, respectively, further validating the effectiveness of the adaptive attention model in the global branch. Finally, after using the pre-trained weights of the Adltformer-enhanced model as a pre-encoder and adding them to the backbone network of the Detr detection model for team-training, the recall rate and mAP50 increased by 1.0% and 1.1%, respectively. These results demonstrate that the team-training of Adltformer and Detr significantly enhances detection accuracy. To evaluate the generalization ability of the AT-Detr model, we trained the algorithm on the public ExDARK dataset. The final recall rate and mAP50 increased by 0.6% and 0.5%, respectively, further confirming the feasibility and effectiveness of the AT-Detr algorithm in low-light image detection tasks.

The analysis shows that the two models, Adltformer and Detr, each perform distinct tasks, with Adltformer focusing on image enhancement and Detr handling target detection. However, through team-training, these two models can share gradients and adjust the parameters of the entire network to minimize the loss function associated with the Detr detection task, optimizing the performance of the network as a whole.

The team-training process goes beyond optimizing each task individually; it improves overall system performance through joint optimization. For instance, when Adltformer enhances low-light images, it not only focuses on improving the quality of local and global features but ensures that the enhanced image is more suitable for target detection using Detr. As a result, the image enhancement task is no longer solely driven by the needs of human vision but optimized to meet the requirements of machine vision, making the enhanced image better suited for subsequent detection. In summary, the training and optimization processes of the entire system are coordinated, allowing the two models to work together to address issues related to loss or the insufficient processing of image detail information, which may occur during separate training. Ultimately, this collaboration enables the system to better adapt to complex tasks in low-light environments.

## 4. Discussion

In the field of low-light image enhancement, acquiring low-light images that are perfectly aligned with normal-light images containing noise is essential. However, traditional acquisition methods are often impractical in this scenario due to the difficulty of achieving precise alignment in real-world scenes (e.g., dynamically moving cows). Therefore, the DTN-Synthesis algorithm proposed in this study removes brightness and adjusts the exposure of real daytime images, simulating nighttime light sources and noise to generate realistic low-light images. These generated image data not only address the problem of insufficient experimental data but enhance the effectiveness of low-light image enhancement model training.

In the design of the enhancement model, the Adltformer model optimizes the image’s overall information-capturing ability by adaptively adjusting ISP parameters. Compared with existing methods, Adltformer achieves superior enhancement with fewer parameters, providing robust support for subsequent target detection. However, challenges remain in detection based solely on augmented images: although the augmented images are more suitable for human observation, the gap between human and machine vision results in suboptimal detection performance [49]. To address this issue, we propose the AT-Detr algorithm. This employs a team-training strategy, enabling the enhancement and detection models to be trained collaboratively to better meet machine vision detection requirements. This method significantly enhances detection accuracy for low-light images and verifies the effectiveness of co-optimization in low-light image detection.

## 5. Conclusions

This study introduced AT-Detr, a novel algorithm that integrates adaptive image enhancement with object detection to address the challenges of cattle detection under complex lighting conditions in real pasture environments. By employing the DTN-Synthesis algorithm, we successfully generated high-quality paired image datasets, and the Adltformer model was utilized to enhance low-light images, improving their visual quality. By leveraging team-training with the Detr detection model, the AT-Detr algorithm can significantly improve detection accuracy and robustness in low-light conditions.

The experimental results show that AT-Detr achieves an over 1% improvement in both the recall and mAP compared with SOTA methods, confirming its effectiveness in detecting cattle under suboptimal lighting conditions. Furthermore, experiments on the ExDARK public dataset highlight the algorithm’s generalization capability, suggesting its potential for application in a broader range of object detection tasks across various low-light environments.

In the future, we plan to further optimize the Adltformer model to better accommodate advanced machine vision tasks while reducing the number of network parameters to enable efficient deployment on edge devices. Additionally, we aim to explore methods that integrate both human and machine vision objectives in image enhancement, thus improving detection accuracy while aligning with human visual perception.

## Figures and Tables

**Table 1:** Detailed composition of experimental data.

Data	Daytime Videos	Nighttime Videos
24 July 2022	59	21
25 July 2022	50	34
26 July 2022	68	40

**Table 2:** Classifications and roles of datasets.

Number and Types of Datasets	Role
823 normal-light images	Composition of image pairs to train the Adltformer model
823 synthetic images	
865 real low-light images	Normal- and low-light detection comparison experiment
865 normal-light images	

**Table 3:** Unenhanced PSNR and SSIM metrics.

Number and Type of Datasets	PSNR	SSIM
823 normal light images	10.228	0.355
823 synthetic images		

**Table 4:** Enhanced processed PSNR and SSIM metrics.

Number and Type of Datasets	PSNR	SSIM
823 normal light images	17.254	0.699
823 enhanced images		

**Table 5:** Analysis of results from different enhancement algorithms.

Methods	Image Quality Metrics		Efficiency Metrics	
	PSNR	SSIM	FPS (f/s)	Params (M)
URetinex-Net [30]	12.518	0.436	1.02	0.63
MiRNet [31]	12.765	0.512	0.20	31.79
StableLLVE [32]	11.276	0.370	6.34	4.32
SNR-Aware [33]	16.636	0.486	2.91	39.12
RQ-LLIE [34]	13.231	0.425	0.12	34.59
RetinexNet [35]	12.551	0.500	0.99	1.84
NeRCo [36]	12.906	0.352	0.23	23.30
KinD [37]	14.150	0.492	0.68	8.16
Adltformer (ours)	17.254	0.699	17.44	0.12

**Table 6:** Detr detection index under different light conditions.

Method	Datasets	Recall/%	mAP50/%
Detr-base	865 low-light images	98.4	96.4
	865 normal-light images	99.0	96.6
	1730 mixed images	98.6	96.0

**Table 7:** Performance comparison of different enhancement algorithms.

Methods	Recall%	mAP50/%
Detr-base [26]	98.4	96.4
SNR-Aware [33]	99.0	97.0
KinD [37]	99.1	96.2
RUAS [38]	95.9	92.2
SCI [39]	98.6	96.2
Zero-DCE++ [40]	98.7	96.7
MBLLEN [41]	98.7	96.8
LMP-GP [42]	99.1	96.6
CSEC [43]	99.0	96.7
HVI-CIDNet [44]	98.7	96.4
Adltformer-Detr	99.1	97.0
AT-Detr (ours)	99.4	97.5

**Table 8:** Detection results for the algorithm based on different ExDARK classes.

Methods	Bicycle	Boat	Bottle	Bus	Car	Cat	Chair	Cup	Dog	Motor	People	Table	mAP/%
Detr-base [26]	75.8	72.8	72.4	93.4	78.5	66.1	66.1	69.2	74.7	66.4	73.9	57.8	72.3
StableLLVE [32]	75.8	73.8	70.6	89.7	78.3	68.9	66.4	68.7	74.6	67.5	74.4	60.6	72.5
RUAS [38]	73.9	70.4	71.0	92.3	78.5	68.4	62.1	64.7	71.5	66.7	72.9	58.0	70.9
SCI [39]	72.5	73.1	70.6	91.4	80.9	68.4	64.2	67.2	75.3	67.6	73.8	58.0	71.9
Zero-DCE++ [40]	73.4	74.1	72.0	92.7	80.6	70.0	64.1	65.6	73.8	69.0	74.3	59.2	72.4
LMP-GP [42]	76.7	66.7	67.0	87.7	61.5	66.0	61.3	64.5	70.2	54.3	64.1	50.2	65.9
CSEC [43]	75.6	69.8	66.8	90.7	63.7	63.3	60.7	66.3	70.6	55.8	65.0	52.5	66.7
HVI-CIDNet [44]	73.9	73.6	68.8	89.6	80.4	66.5	66.3	67.3	75.4	66.7	73.8	59.6	71.8
ChebyLighter [46]	69.1	68.3	67.8	86.0	72.5	64.5	62.4	56.7	70.4	58.1	67.7	54.8	66.5
EFINet [47]	73.3	71.5	65.8	86.8	65.1	58.6	60.8	64.4	75.6	55.5	64.6	50.8	66.0
Adltformer-Detr	76.1	70.8	73.8	91.8	80.5	68.1	63.9	67.6	76.4	67.2	72.6	60.1	72.4
AT-Detr	77.3	71.7	73.7	91.7	81.2	70.0	65.0	68.0	73.4	67.2	74.6	59.7	72.8

**Table 9:** Ablation test evaluation results for Adltformer image enhancement algorithm. The symbol √ indicates that this module was added during the model training process.

Local Branch	Layer Norm	Color Norm	Global Adaptive Attention	Global WB	Global CCM	Global Gamma	PSNR	SSIM
							10.228	0.355
√	√						12.451	0.393
√		√					13.169	0.457
√		√	√				15.930	0.636
√		√	√	√			16.336	0.661
√		√	√	√	√		16.717	0.680
√		√	√	√	√	√	17.254	0.699

**Table 10:** Ablation test evaluation results for AT-Detr target detection algorithm. The symbol √ indicates that this module was added during the model training process.

Datasets	Detr	Adltformer Local Branch	Adltformer Global Branch	Team-Training	Recall/%	mAP50/%
Cow-night	√				98.4	96.4
	√	√			98.6 (+0.4)	96.7 (+0.3)
	√	√	√		99.1(+0.7)	97.0 (+0.6)
	√	√	√	√	**99.4 (+1.0)**	**97.5 (+1.1)**
ExDARK	√				90.1	72.3
	√	√			90.2 (+0.1)	72.3 (+0.0)
	√	√	√		90.4 (+0.3)	72.4 (+0.1)
	√	√	√	√	**90.7 (+0.6)**	**72.8 (+0.5)**

## Data Availability

The authors do not have permission to share the data.
